# Elevated serum plasminogen activator inhibitor-1 is associated with seizure burden, drug resistance, and neuroinflammatory markers in pediatric epilepsy

**DOI:** 10.3389/fped.2026.1804857

**Published:** 2026-07-14

**Authors:** Xiao Xiao, Xiaoyan Shi, Jun Feng, Xinyu Zhou, Manli Wang, Bingbing Zhang, Chen Xu, Jihong Tang

**Affiliations:** Children’s Hospital of Soochow University, Suzhou, China

**Keywords:** biomarker, blood-Brain barrier, neuroinflammation, PAI-1, pediatric epilepsy, plasminogen activator inhibitor-1, seizure susceptibility

## Abstract

**Background:**

Neuroinflammation and blood-brain barrier (BBB) disruption are increasingly recognized as key pathophysiological mechanisms in epilepsy. Plasminogen Activator Inhibitor-1 (PAI-1), a critical regulator of fibrinolysis and extracellular matrix remodeling, has been implicated in inflammatory processes, but its specific role in pediatric epilepsy remains largely unexplored. This study aimed to investigate the association of PAI-1 with neuroinflammation, seizure susceptibility, and clinical outcomes in a cohort of pediatric epilepsy patients.

**Methods:**

In this case-control study, we enrolled 200 pediatric patients with epilepsy and 100 age- and sex-matched healthy controls. Serum levels of PAI-1, pro- and anti-inflammatory cytokines (including IL-6, IL-1β, TNF-α), and markers of BBB disruption and neuronal injury (S100B, NSE, GFAP) were quantified using enzyme-linked immunosorbent assays (ELISA). Comprehensive clinical data, including seizure frequency, epilepsy type, and treatment response, were collected. Statistical analyses, including *t*-tests, ANOVA, Pearson correlation, and multivariate linear regression, were performed to assess group differences and identify predictors of seizure severity. Receiver Operating Characteristic (ROC) analysis was used to evaluate the diagnostic performance of PAI-1.

**Results:**

Serum PAI-1 levels were significantly elevated in epilepsy patients compared to healthy controls (28.47 ± 12.10 ng/mL vs. 11.35 ± 5.05 ng/mL; *p* < 0.001). PAI-1 concentrations demonstrated a strong positive correlation with seizure frequency (*r* = 0.537, *p* < 0.001) and were significantly higher in patients with drug-resistant epilepsy (*p* < 0.001). ROC analysis revealed that PAI-1 is an excellent diagnostic biomarker for epilepsy (AUC = 0.916). Among all variables included in the model, PAI-1 showed the strongest independent association with monthly seizure frequency (*β* = 10.79, 95% CI: 8.30–13.28, *p* < 0.001), supporting its role as a biomarker of seizure burden rather than a standalone clinical predictor of seizure occurrence. Furthermore, PAI-1 levels were significantly correlated with markers of neuroinflammation (IL-6: *r* = 0.270, *p* < 0.001) and BBB disruption (S100B: *r* = 0.271, *p* < 0.001). Higher PAI-1 levels were also associated with poorer quality of life scores (*r* = −0.227, *p* < 0.001).

**Conclusion:**

PAI-1 is significantly elevated in pediatric epilepsy and is associated with seizure burden, neuroinflammatory activation, BBB disruption, drug resistance, and poorer quality of life. In this clinical setting, PAI-1 may be more useful as a risk-stratification biomarker for identifying children with higher disease burden and possible treatment resistance, rather than as a standalone tool for predicting seizure occurrence. Longitudinal studies are needed to determine whether baseline or dynamic changes in PAI-1 can predict AED response, treatment escalation, and progression toward drug-resistant epilepsy.

## 1 Introduction

Epilepsy is one of the most common chronic neurological disorders affecting children and adolescents, with a global prevalence of approximately 0.9% (1). The burden of pediatric epilepsy extends beyond seizure control, significantly impacting cognitive development, psychosocial well-being, and overall quality of life for both patients and their families (2). Despite the availability of numerous antiepileptic drugs (AEDs), nearly one-third of children with epilepsy develop drug-resistant epilepsy (DRE), highlighting the urgent need for a deeper understanding of the underlying pathophysiology to identify novel therapeutic targets and biomarkers (3).

In recent years, a paradigm shift has occurred in our understanding of epileptogenesis, moving beyond the concept of epilepsy as a purely neuronal disorder to one involving a complex interplay of glial cells, the neurovascular unit, and the immune system (4). A growing body of evidence implicates neuroinflammation and the associated breakdown of the blood-brain barrier (BBB) as key drivers in the initiation and perpetuation of seizures (5). Pro-inflammatory cytokines, such as Interleukin-1 beta (IL-1β), Interleukin-6 (IL-6), and Tumor Necrosis Factor-alpha (TNF-α), are consistently found to be elevated in the brain and peripheral circulation of epilepsy patients (6). These molecules can increase neuronal excitability, lower the seizure threshold, and contribute to the cycle of inflammation and neuronal injury that characterizes the epileptic brain (7).

The plasminogen activation system, a crucial enzymatic cascade involved in fibrinolysis, extracellular matrix remodeling, and cell migration, has emerged as a significant player at the interface of vascular homeostasis and neuroinflammation (8). Plasminogen Activator Inhibitor-1 (PAI-1), encoded by the SERPINE1 gene, is the primary physiological inhibitor of tissue-type (tPA) and urokinase-type (uPA) plasminogen activators (9). While essential for regulating fibrinolysis, excessive PAI-1 activity has been linked to a range of pathological conditions, including cardiovascular disease, thrombosis, and chronic inflammatory states (10). In the central nervous system (CNS), PAI-1 is produced by various cell types, including endothelial cells, astrocytes, and microglia, and its expression is potently induced by inflammatory stimuli. Elevated PAI-1 levels have been associated with BBB dysfunction, microvascular complications, and pro-inflammatory signaling in various neurological disorders, including stroke and Alzheimer's disease (11, 12).

Despite the compelling evidence linking PAI-1 to both inflammation and vascular dysfunction, two core components of modern epilepsy pathophysiology, its specific role in pediatric epilepsy remains largely uninvestigated (13). The intricate connections between the plasminogen system, neuroinflammation, and seizure susceptibility present a critical knowledge gap (14). We hypothesized that PAI-1 is upregulated in pediatric epilepsy and that its levels correlate with the degree of neuroinflammation, seizure severity, and clinical outcomes (15, 16). Identifying PAI-1 as a key player in this process could unveil a novel biomarker for disease activity and a potential therapeutic target for mitigating neuroinflammation and improving seizure control (17, 18).

Therefore, this study aimed to comprehensively investigate the role of PAI-1 in a well-characterized cohort of pediatric epilepsy patients. The primary objectives were: (1) to compare serum PAI-1 levels between pediatric epilepsy patients and healthy controls; (2) to determine the association of PAI-1 with seizure frequency, drug resistance, and other clinical parameters; (3) to explore the correlation between PAI-1 and key markers of neuroinflammation and BBB disruption; and (4) to evaluate the potential of PAI-1 as an independent predictor of seizure susceptibility and its impact on quality of life.

## 2 Materials and methods

### 2.1 Study design and participants

This prospective case-control study was conducted at the Department of Pediatric Neurology between January 2023 and December 2024. The study enrolled 200 pediatric patients diagnosed with epilepsy and 100 age- and sex-matched healthy controls. Epilepsy was diagnosed according to the International League Against Epilepsy (ILAE) 2017 classification criteria based on clinical history, electroencephalography (EEG), and neuroimaging findings. Drug-resistant epilepsy (DRE) was defined as failure to achieve sustained seizure freedom after adequate trials of two tolerated and appropriately chosen antiepileptic drug (AED) schedules, whether as monotherapy or in combination.

Inclusion criteria for the epilepsy group were: (1) age between 1 and 18 years; (2) confirmed diagnosis of epilepsy with at least two unprovoked seizures occurring more than 24 h apart; and (3) availability of complete clinical and laboratory data. Exclusion criteria included: (1) acute infections or chronic inflammatory diseases; (2) autoimmune or metabolic disorders; (3) use of immunosuppressive or anti-inflammatory medications within the preceding four weeks; (4) history of neurosurgical intervention; and (5) progressive neurological conditions. Healthy controls were recruited from children attending routine health check-ups with no history of neurological disorders, seizures, or chronic illnesses.

The study protocol was approved by the Institutional Ethics Committee and conducted in accordance with the Declaration of Helsinki. Written informed consent was obtained from parents or legal guardians, and assent was obtained from participants aged 7 years and older.

### 2.2 Clinical data collection

Comprehensive clinical data were collected through structured interviews, medical record review, and standardized assessments. Demographic variables included age, sex, body mass index (BMI), and socioeconomic status. Epilepsy-specific variables included epilepsy type, seizure type, epilepsy syndrome, etiology, age at seizure onset, duration of epilepsy, seizure frequency (categorized and per month), history of status epilepticus, and current AED regimen. Drug resistance status was determined based on ILAE criteria. EEG findings were documented, including background activity, presence and frequency of epileptiform discharges, and localization. Neuroimaging results from magnetic resonance imaging (MRI) were recorded, noting the presence of structural abnormalities, lesion type, and hippocampal sclerosis.

Quality of life was assessed using the Quality of Life in Childhood Epilepsy (QOLCE) questionnaire and the Pediatric Quality of Life Inventory (PedsQL) Generic Core Scales, both validated for use in pediatric populations.

### 2.3 Sample collection and processing

Venous blood samples (5 mL) were collected from all participants after an overnight fast (minimum 8 h) between 8:00 and 10:00 AM to minimize circadian variation. For epilepsy patients, samples were obtained during the interictal period, at least 24 h after the last seizure event. Blood was collected into serum separator tubes and allowed to clot at room temperature for 30 min. Samples were then centrifuged at 3,000 rpm for 15 min, and serum was aliquoted and stored at −80 °C until analysis.

In a subset of patients (*n* = 50) who underwent lumbar puncture for clinical indications, cerebrospinal fluid (CSF) samples were collected and processed similarly for analysis of CSF biomarkers.

### 2.4 Laboratory measurements

Serum levels of PAI-1 were measured using a commercially available enzyme-linked immunosorbent assay (ELISA) kit (Human PAI-1 ELISA, R&D Systems, Minneapolis, MN, USA) according to the manufacturer's instructions. The assay had a sensitivity of 0.1 ng/mL and intra- and inter-assay coefficients of variation of less than 5% and 8%, respectively.

Pro-inflammatory cytokines (IL-1β, IL-6, TNF-α, IL-8, IFN-*γ*) and anti-inflammatory cytokines (IL-10, TGF-β1) were quantified using multiplex bead-based immunoassays (Bio-Plex Pro Human Cytokine Panel, Bio-Rad Laboratories, Hercules, CA, USA). High-sensitivity C-reactive protein (hs-CRP) was measured by immunoturbidimetric assay on an automated analyzer.

Markers of blood-brain barrier disruption and neuronal injury, including S100 calcium-binding protein B (S100B), neuron-specific enolase (NSE), and glial fibrillary acidic protein (GFAP), were measured using commercial ELISA kits (Millipore, Burlington, MA, USA). Components of the plasminogen activation system, including tissue-type plasminogen activator (tPA), urokinase-type plasminogen activator (uPA), D-dimer, fibrinogen, and plasminogen activity, were also measured using standard laboratory methods.

All assays were performed in duplicate by trained laboratory personnel blinded to the clinical status of participants.

### 2.5 Statistical analysis

Statistical analyses were performed using SPSS version 26.0 (IBM Corp., Armonk, NY, USA) and Python 3.11 with SciPy and Statsmodels libraries. Continuous variables were expressed as mean ± standard deviation (SD) or median with interquartile range (IQR) depending on data distribution, assessed by the Shapiro–Wilk test. Categorical variables were presented as frequencies and percentages.

Comparisons between two groups were performed using independent samples *t*-test for normally distributed continuous variables and Mann–Whitney *U* test for non-normally distributed data. Comparisons among multiple groups were conducted using one-way analysis of variance (ANOVA) with Tukey's *post-hoc* test or Kruskal–Wallis test as appropriate. Categorical variables were compared using chi-square test or Fisher's exact test.

Correlations between continuous variables were assessed using Pearson's correlation coefficient for normally distributed data and Spearman's rank correlation coefficient for non-parametric data. Multivariate linear regression analysis was performed to identify independent predictors of seizure frequency, with variables showing *p* < 0.1 in univariate analysis entered into the model. Standardized beta coefficients with 95% confidence intervals were reported.

Receiver Operating Characteristic (ROC) curve analysis was performed to evaluate the diagnostic performance of PAI-1 in distinguishing epilepsy patients from healthy controls. The area under the curve (AUC), sensitivity, specificity, and optimal cutoff value (determined by Youden's *J* statistic) were calculated. In addition to the epilepsy-vs.-control ROC analysis, a subgroup ROC analysis was performed to evaluate the ability of serum PAI-1 to distinguish well-controlled epilepsy from drug-resistant epilepsy. Well-controlled epilepsy was defined as non-drug-resistant epilepsy with seizure freedom for more than 12 months. Drug-resistant epilepsy was considered the positive classification group. The optimal cutoff value was determined using Youden's *J* statistic. Unsupervised clustering was tested as an exploratory analysis within the epilepsy group, to see if patients could be clustered on the basis of biomarker profile and clinical severity. A blood marker of PAI-1, inflammatory cytokines, BBB/neuronal injury markers, seizure frequency, the number of AEDs used and quality-of-life scores were included. Before clustering, all the continuous variables were standardized. Patterns were analysed descriptively and presented graphically by principal component analysis. Clusters were subsequently compared in terms of seizure burden, PAI-1 levels, BBB/neuronal injury markers, AED burden, and quality-of-life measures.

A two-tailed *p*-value of less than 0.05 was considered statistically significant for all analyses.

## 3 Results

### 3.1 Participant characteristics

The study enrolled a total of 300 participants, comprising 200 pediatric patients diagnosed with epilepsy and 100 age- and sex-matched healthy controls. The demographic and clinical characteristics of the study population are summarized in Table 1. The mean age was comparable between the epilepsy group (9.32 ± 3.81 years) and the control group (9.23 ± 3.85 years), with no statistically significant difference observed (*p* = 0.847). The sex distribution was balanced in both groups, with females constituting 49.5% of the epilepsy cohort and 52.0% of the control group. Body mass index (BMI) was also similar between the two groups (18.40 ± 3.16 kg/m^2^ vs. 18.12 ± 3.76 kg/m^2^; *p* = 0.502).

**Table 1:** Demographic and clinical characteristics of study participants.

Characteristic	Control Group (*n* = 100)	Epilepsy Group (*n* = 200)	*p*-value
Demographics			
Age (years), mean ± SD	9.23 ± 3.85	9.32 ± 3.81	0.847
Sex, *n* (%)			0.681
Female	52 (52.0%)	99 (49.5%)	
Male	48 (48.0%)	101 (50.5%)	
BMI (kg/m^2^), mean ± SD	18.12 ± 3.76	18.40 ± 3.16	0.502
Clinical Characteristics			
Age at Onset (years), mean ± SD	—	4.45 ± 3.77	—
Duration of Epilepsy (years), mean ± SD	—	2.67 ± 2.50	—
Seizure Frequency (per month), mean ± SD	—	10.44 ± 19.14	—
Epilepsy Type, *n* (%)	—		—
Focal		94 (47.0%)	
Generalized		68 (34.0%)	
Combined Focal-Generalized		19 (9.5%)	
Unknown		19 (9.5%)	
Drug-Resistant Epilepsy, *n* (%)	—	58 (29.0%)	—
Number of AEDs, mean ± SD	—	1.78 ± 1.16	—

SD, standard deviation; BMI, body mass index; AEDs, antiepileptic drugs. *p*-values were calculated using independent samples *t*-test for continuous variables and chi-square test for categorical variables.

Within the epilepsy cohort, the mean age at seizure onset was 4.45 ± 3.77 years, with a mean disease duration of 2.67 ± 2.50 years at the time of enrollment. The median seizure frequency was 4 seizures per month, though this varied considerably across the cohort (range: 0 to 90 per month). Focal epilepsy was the most prevalent epilepsy type, accounting for 47.0% of cases (*n* = 94), followed by generalized epilepsy at 34.0% (*n* = 68). Combined focal-generalized epilepsy and unknown epilepsy types each represented 9.5% of the cohort (*n* = 19 each). Notably, drug-resistant epilepsy (DRE), defined according to the International League Against Epilepsy (ILAE) criteria as failure of adequate trials of two tolerated and appropriately chosen antiepileptic drug schedules, was identified in 29.0% (*n* = 58) of the patients. The mean number of antiepileptic drugs (AEDs) prescribed was 1.78 ± 1.16, reflecting the varied treatment complexity within the cohort.

### 3.2 Elevated PAI-1 levels in pediatric epilepsy patients

**Figure 1:**
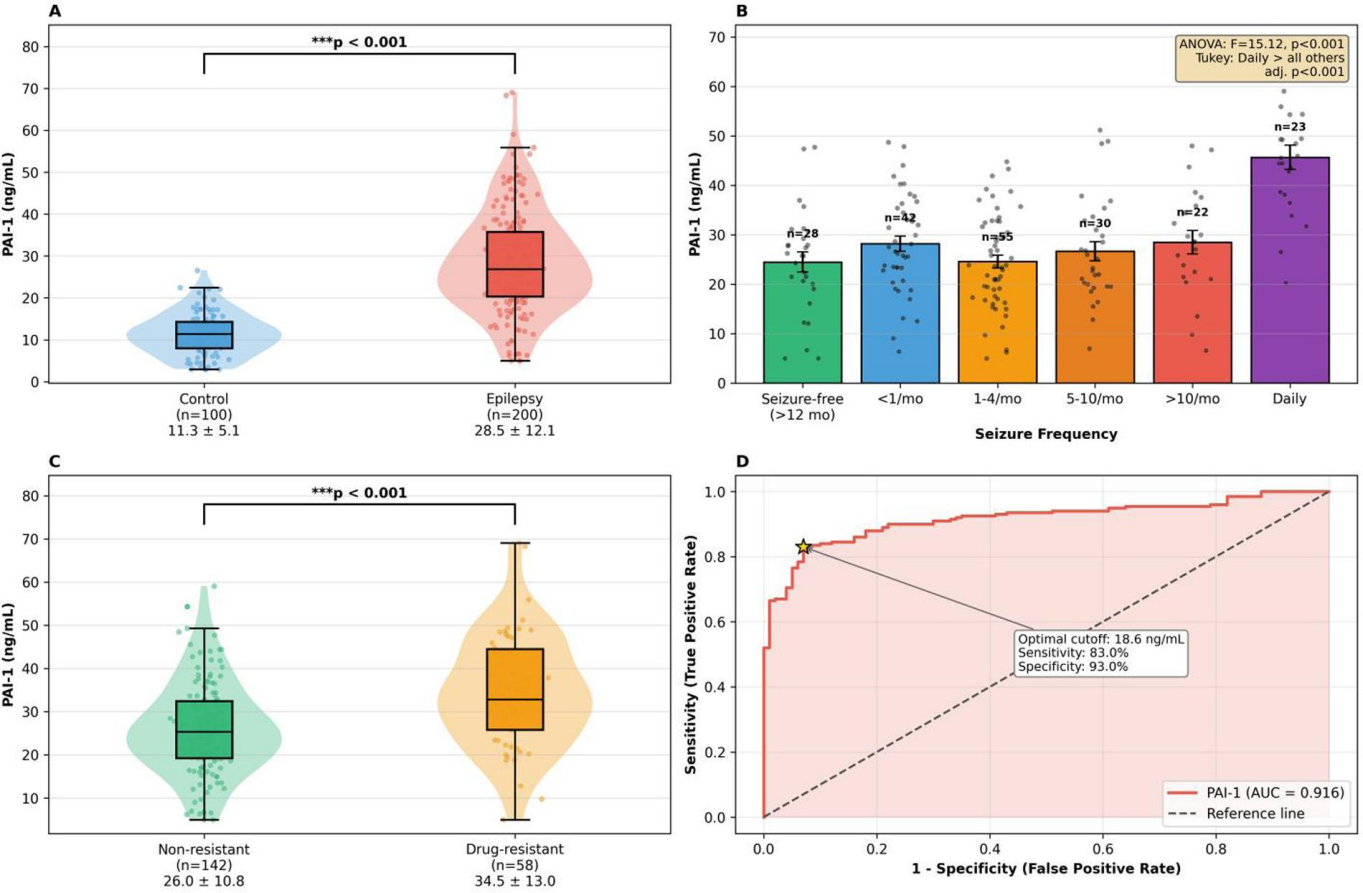
PAI-1 levels in pediatric epilepsy patients. **(A)** Violin and box plots showing significantly higher serum PAI-1 levels in epilepsy patients compared to healthy controls. Individual data points are overlaid on the plots. **(B)** Bar chart demonstrating a progressive increase in PAI-1 levels with higher seizure frequency categories. Error bars represent standard error of the mean (SEM). **(C)** Violin and box plots indicating significantly elevated PAI-1 in patients with drug-resistant epilepsy compared to non-resistant patients. **(D)** Receiver Operating Characteristic (ROC) curve analysis showing the diagnostic performance of PAI-1 for distinguishing epilepsy patients from controls. The optimal cutoff point is marked with a star.

Serum levels of Plasminogen Activator Inhibitor-1 (PAI-1) were significantly elevated in pediatric patients with epilepsy compared to healthy controls. The mean PAI-1 concentration in the epilepsy group was 28.47 ± 12.10 ng/mL, which was more than 2.5-fold higher than the control group (11.35 ± 5.05 ng/mL; *p* < 0.001), as illustrated in Figure 1A. This substantial difference highlights a pronounced dysregulation of the plasminogen activation system in the patient cohort and suggests a potential role for PAI-1 in the pathophysiology of pediatric epilepsy.

We further investigated the association between PAI-1 levels and seizure activity. A clear, graded relationship was observed between PAI-1 concentrations and seizure frequency categories. Patients who were seizure-free for more than 12 months had the lowest mean PAI-1 levels (24.8 ng/mL), while those experiencing daily seizures had the highest (45.2 ng/mL). PAI-1 levels differed significantly across seizure-frequency categories (one-way ANOVA, F = 15.12, *p* < 0.001; Figure 1B). *Post-hoc* Tukey comparisons showed that this overall effect was primarily driven by the daily-seizure group, which had significantly higher PAI-1 levels than the other seizure-frequency groups. In contrast, most non-daily seizure-frequency groups did not differ significantly from each other after correction for multiple comparisons. These findings indicate that the strongest PAI-1 elevation was observed in patients with the highest seizure burden. Furthermore, patients with drug-resistant epilepsy (DRE) exhibited significantly higher PAI-1 levels compared to those with non-resistant epilepsy (34.49 ± 13.00 ng/mL vs. 26.01 ± 10.80 ng/mL; *p* < 0.001; Figure 1C), suggesting that PAI-1 elevation may be associated with a more refractory disease course. The *p*-value shown in Figure 1C reflects the direct comparison between non-drug-resistant and drug-resistant epilepsy groups. To further evaluate the clinical relevance of PAI-1 for treatment-related stratification, we performed an additional ROC analysis comparing well-controlled epilepsy with drug-resistant epilepsy. Well-controlled epilepsy was defined as non-drug-resistant epilepsy with seizure freedom for more than 12 months. In this analysis, serum PAI-1 demonstrated moderate discriminatory performance for distinguishing drug-resistant epilepsy from well-controlled epilepsy, with an AUC of 0.742. The optimal cutoff value was 32.0 ng/mL, yielding a sensitivity of 58.6% and specificity of 89.5%. These findings suggest that PAI-1 may be more clinically informative as a marker of treatment resistance or disease burden than as a standalone diagnostic marker of epilepsy (Supplementary Figure S1). To evaluate the diagnostic utility of PAI-1 as a biomarker for distinguishing epilepsy patients from healthy controls, a Receiver Operating Characteristic (ROC) curve analysis was performed. PAI-1 demonstrated excellent discriminatory performance, with an Area Under the Curve (AUC) of 0.916 (95% CI: 0.882–0.950). An optimal cutoff value of 18.6 ng/mL was identified using Youden's *J* statistic, yielding a sensitivity of 83.0% and a specificity of 93.0% (Figure 1D). These findings suggest that PAI-1 distinguishes children with epilepsy from healthy controls in this cohort; however, this result should be interpreted as preliminary because clinically relevant neurological disease control groups were not included.

### 3.3 Pro-inflammatory cytokine profile in epilepsy patients

To characterize the neuroinflammatory state associated with pediatric epilepsy, we measured a comprehensive panel of pro- and anti-inflammatory cytokines. The epilepsy group exhibited a distinct pro-inflammatory profile, with significantly elevated serum levels of multiple cytokines compared to the control group. The detailed comparison of inflammatory markers between groups is presented in Table 2.

**Table 2:** Comparison of inflammatory and neuronal injury markers between groups.

Biomarker	Control Group (*n* = 100)	Epilepsy Group (*n* = 200)	Fold Change	*p*-value
Inflammatory Markers				
IL-1β (pg/mL)	1.83 ± 0.85	5.33 ± 2.45	2.91×	<0.001
IL-6 (pg/mL)	2.45 ± 1.11	9.15 ± 4.01	3.73×	<0.001
TNF-α (pg/mL)	3.46 ± 1.32	8.35 ± 3.47	2.42×	<0.001
IL-8 (pg/mL)	11.38 ± 4.52	24.69 ± 9.87	2.17×	<0.001
IFN-*γ* (pg/mL)	6.05 ± 2.41	10.69 ± 4.28	1.77×	<0.001
IL-10 (pg/mL)	4.59 ± 1.87	6.30 ± 2.89	1.37×	<0.001
TGF-β1 (ng/mL)	9.69 ± 3.88	14.17 ± 5.67	1.46×	<0.001
hs-CRP (mg/L)	0.89 ± 0.63	2.02 ± 1.21	2.28×	<0.001
Neuronal Injury Markers				
S100B (ng/mL)	0.037 ± 0.034	0.117 ± 0.079	3.19×	<0.001
NSE (ng/mL)	8.26 ± 1.90	12.79 ± 4.10	1.55×	<0.001
GFAP (ng/mL)	0.026 ± 0.026	0.065 ± 0.068	2.49×	<0.001

Data are presented as mean ± standard deviation. Fold change is calculated as Epilepsy/Control. *p*-values were calculated using independent samples *t*-test. IL, interleukin; TNF-α, tumor necrosis factor-alpha; IFN-γ, interferon-gamma; TGF-β1, transforming growth factor-beta 1; hs-CRP, high-sensitivity C-reactive protein; S100B, S100 calcium-binding protein B; NSE, neuron-specific enolase; GFAP, glial fibrillary acidic protein.

**Figure 2:**
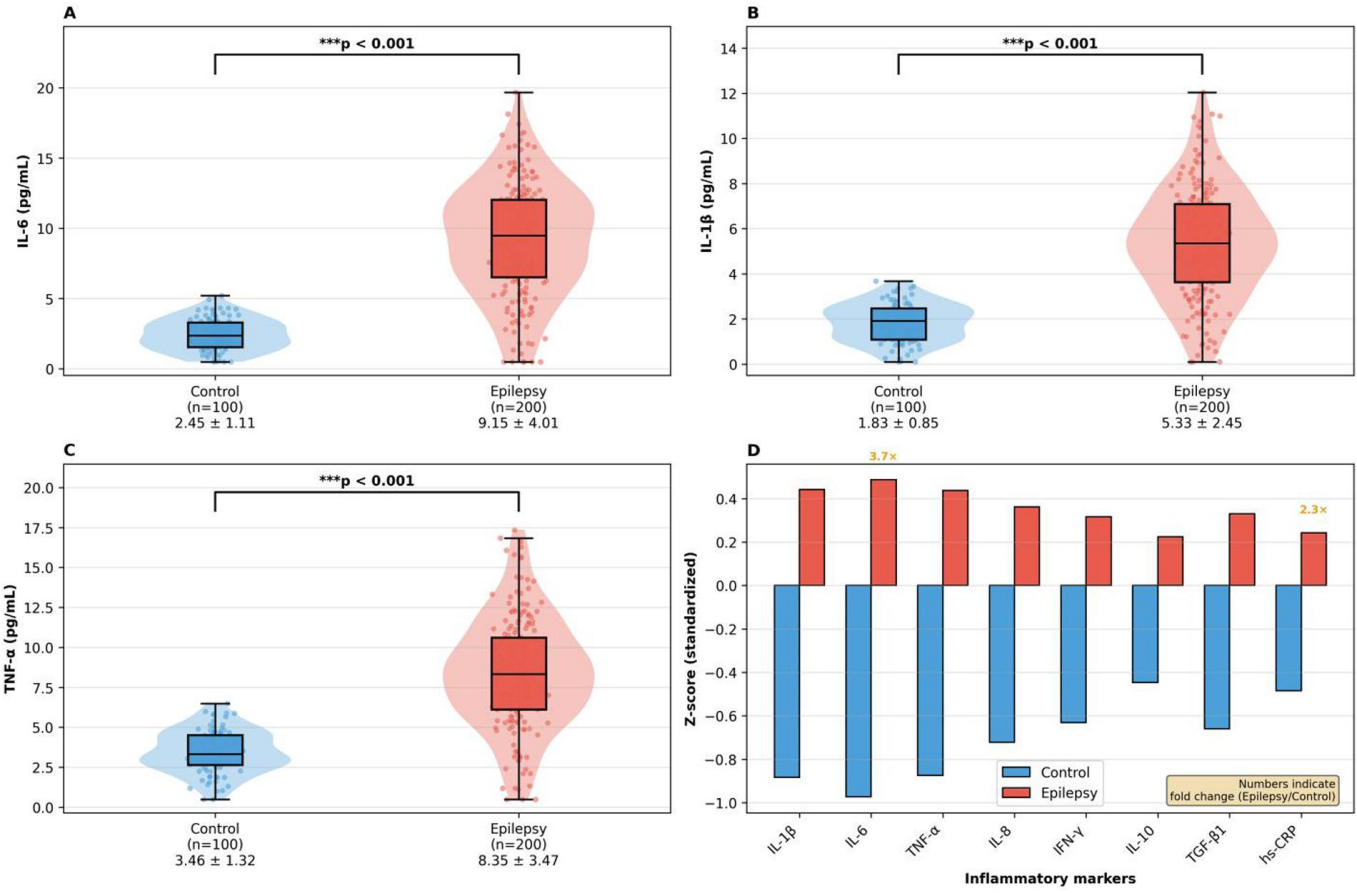
Neuroinflammatory profile in pediatric epilepsy. **(A–C)** Violin and box plots comparing serum levels of IL-6, IL-1β, and TNF-α between epilepsy patients and healthy controls, demonstrating significant elevations in the patient group. **(D)** Standardized Z-score comparison of key inflammatory markers between groups, with fold change annotations indicating the magnitude of increase in the epilepsy group relative to controls.

Interleukin-6 (IL-6) showed the most pronounced elevation, with a 3.73-fold increase in the epilepsy group (9.15 ± 4.01 pg/mL vs. 2.45 ± 1.11 pg/mL; *p* < 0.001; Figure 2A). Interleukin-1 beta (IL-1β) demonstrated a 2.91-fold increase (5.33 ± 2.45 pg/mL vs. 1.83 ± 0.85 pg/mL; *p* < 0.001; Figure 2B), while Tumor Necrosis Factor-alpha (TNF-α) showed a 2.42-fold increase (8.35 ± 3.47 pg/mL vs. 3.46 ± 1.32 pg/mL; *p* < 0.001; Figure 2C). Other inflammatory markers, including IL-8, IFN-*γ*, and high-sensitivity C-reactive protein (hs-CRP), were also significantly elevated in the epilepsy cohort. Notably, even the anti-inflammatory cytokine IL-10 was elevated in the epilepsy group (6.30 ± 2.89 pg/mL vs. 4.59 ± 1.87 pg/mL; *p* < 0.001), possibly reflecting a compensatory response to the heightened inflammatory state. A standardized Z-score comparison of all inflammatory markers visually confirmed the systemic inflammatory burden in the epilepsy cohort (Figure 2D).

### 3.4 Correlation of PAI-1 with seizure activity and inflammatory markers

**Figure 3:**
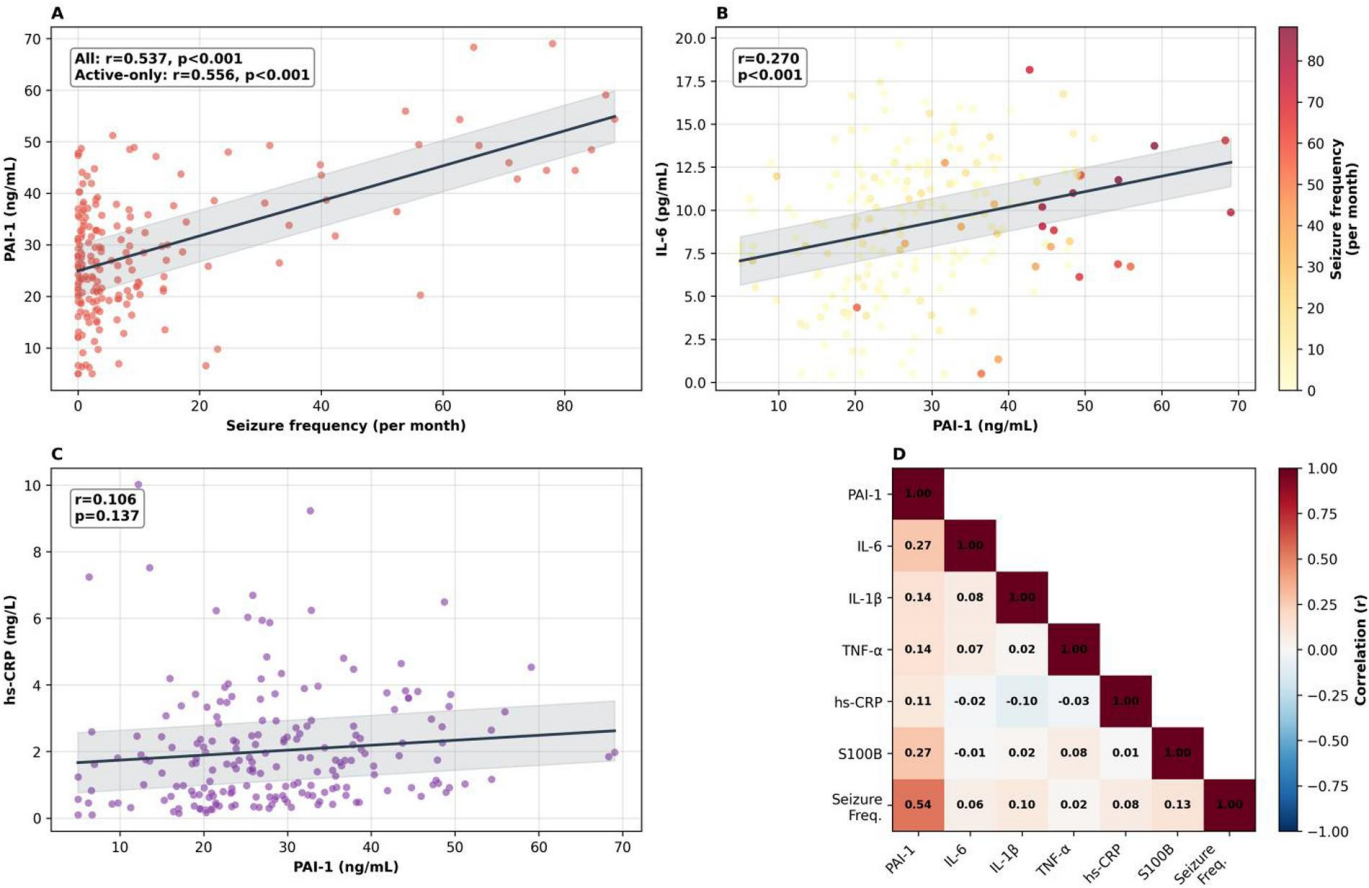
PAI-1 correlations with seizure activity and inflammatory markers. **(A)** Scatter plot showing a strong positive correlation between PAI-1 levels and seizure frequency per month in epilepsy patients. **(B)** Scatter plot demonstrating a significant positive correlation between PAI-1 and IL-6, with data points colored according to seizure frequency to illustrate the interplay between these variables. **(C)** Scatter plot of PAI-1 vs. hs-CRP. **(D)** Correlation matrix heatmap displaying Pearson correlation coefficients among key biomarkers in the epilepsy group. Regression lines with 95% confidence intervals are shown in panels A–C.

We next explored the relationships between PAI-1, seizure severity, and neuroinflammatory markers within the epilepsy cohort. A strong, statistically significant positive correlation was observed between serum PAI-1 levels and monthly seizure frequency (Pearson *r* = 0.537, *p* < 0.001; Figure 3A). To determine whether the association between PAI-1 and seizure frequency was driven by seizure-free or well-controlled patients, we repeated the correlation analysis after excluding these participants. The association between PAI-1 and seizure frequency remained significant among patients with active seizures, indicating that the relationship was not solely explained by inclusion of seizure-free or well-controlled epilepsy cases.

This finding suggests that higher circulating PAI-1 concentrations are associated with more frequent seizure events, potentially implicating PAI-1 in the mechanisms underlying seizure susceptibility.

Furthermore, PAI-1 levels were significantly correlated with several pro-inflammatory cytokines. The strongest correlation was observed with IL-6 (*r* = 0.270, *p* < 0.001; Figure 3B), where the scatter plot, with points colored by seizure frequency, illustrates the complex interplay between PAI-1, inflammation, and seizure burden. Significant correlations were also found with IL-1β (*r* = 0.142, *p* = 0.046) and the neuronal injury marker S100B (*r* = 0.271, *p* < 0.001). The correlation between PAI-1 and hs-CRP was positive but did not reach statistical significance in this cohort (*r* = 0.106, *p* = 0.137; Figure 3C).

A comprehensive correlation matrix heatmap was constructed to visualize the network of relationships among key biomarkers (Figure 3D). This analysis reinforced the strong link between PAI-1 and seizure frequency (*r* = 0.54) and highlighted its connections to inflammatory and injury markers. Notably, seizure frequency itself showed modest correlations with S100B (*r* = 0.13) and hs-CRP (*r* = 0.08), suggesting that PAI-1 may serve as a more sensitive indicator of seizure burden than traditional inflammatory markers alone.

### 3.5 PAI-1 as a biomarker of seizure burden and treatment complexity

A multivariate linear regression analysis was carried out with seizure frequency per month as the dependent variable to determine the factors that influence the frequency of seizures. The model comprised PAI-1, inflammatory markers (IL-6, IL-1b, and TNFa), the biomarker of neuronal injury S100B, and clinical variables (age and duration of epilepsy). The overall model was statistically significant with an *R*^2^ of 0.307 and an F of 12.34 (*p* < 0.001).

**Figure 4:**
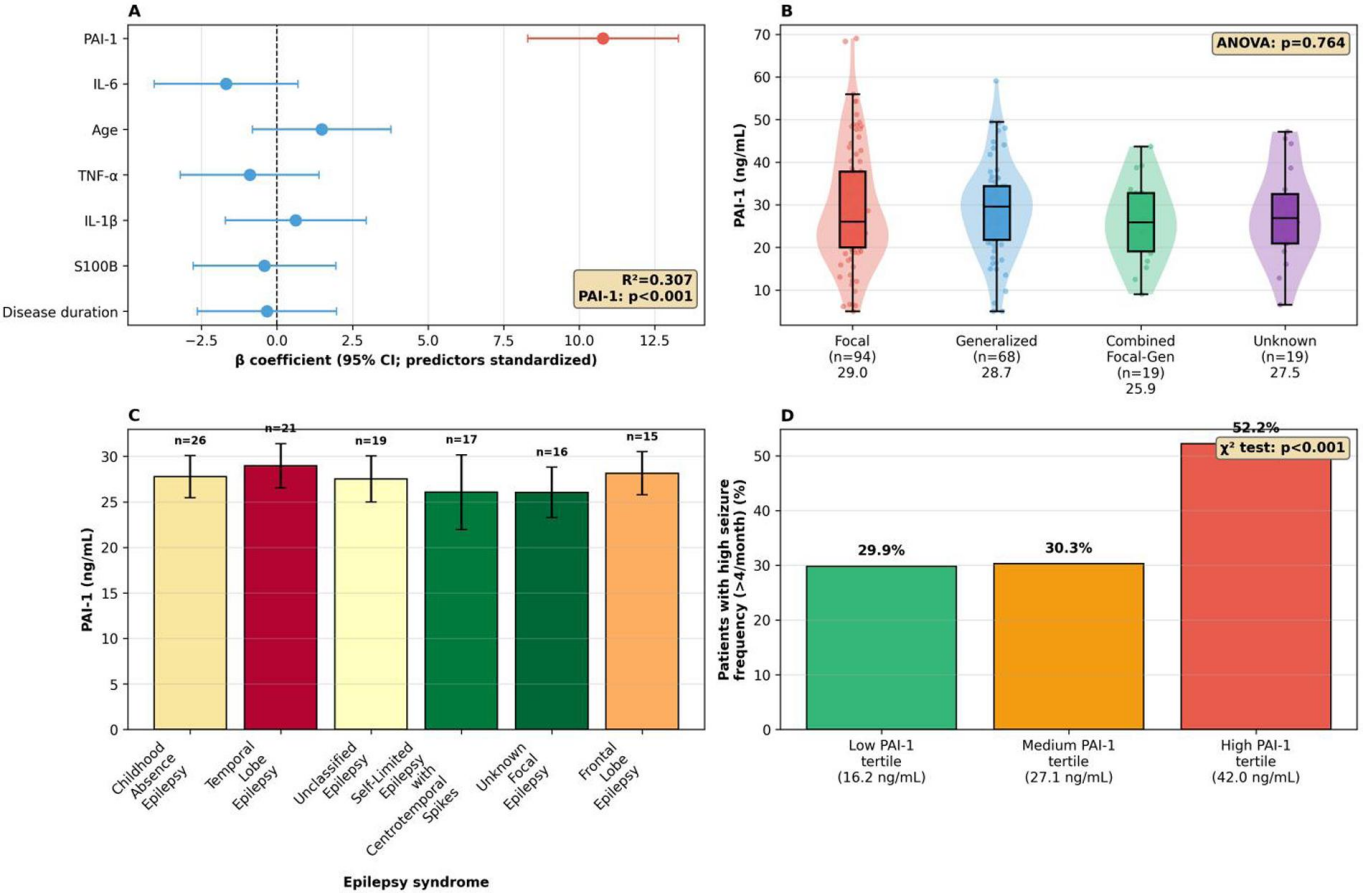
Predictors of seizure susceptibility and PAI-1 distribution. **(A)** Forest plot displaying standardized beta coefficients with 95% confidence intervals from a multivariate linear regression model predicting seizure frequency. PAI-1 is the only significant predictor. **(B)** Violin and box plots showing PAI-1 distribution across different epilepsy types. **(C)** Bar chart comparing PAI-1 levels among the six most frequent epilepsy syndromes. **(D)** Bar chart illustrating the percentage of patients with high seizure frequency (>4/month) across PAI-1 tertiles, demonstrating a significant dose-response relationship.

Among all variables included in the model, PAI-1 showed the strongest independent association with monthly seizure frequency (*β* = 10.79, 95% CI: 8.30–13.28, *p* < 0.001; Figure 4A), supporting its role as a biomarker of seizure burden rather than a standalone clinical predictor of seizure occurrence. Interestingly, there were no significant differences between the major epilepsy types (focal, generalized, combined and unknown; one-way ANOVA, *p* = 0.764; Figure 4B) and the most common epilepsy syndromes (Figure 4C) in terms of PAI-1. This discovery suggests that there is no specific epilepsy type, but rather a more general link between PAI-1 and seizure frequency in the diverse groups of people with epilepsy.

Further, the patients were divided into tertiles according to the serum PAI-1 concentration to illustrate the clinical relevance of PAI-1. There was a remarkable relationship between the tertiles of PAI-1 and proportion of patients with high seizure frequency (more than 4 seizures per month). 29.9% of patients in the lowest tertile of PAI-1 (16.2 ng/mL) experienced a high frequency of seizures. This proportion went up to 30.3% in the upper middle tertile (mean: 27.1 ng/mL) and jumped significantly to 52.2% in the highest tertile (mean: 42.0 ng/mL). This trend was present and statistically significant (*χ*^2^ = 11.8, *p* < 0.001; Figure 4D), solidifying PAI-1's status as a clinically useful seizure susceptibility marker.

### 3.6 Association with blood-brain barrier disruption and neuronal injury

**Figure 5:**
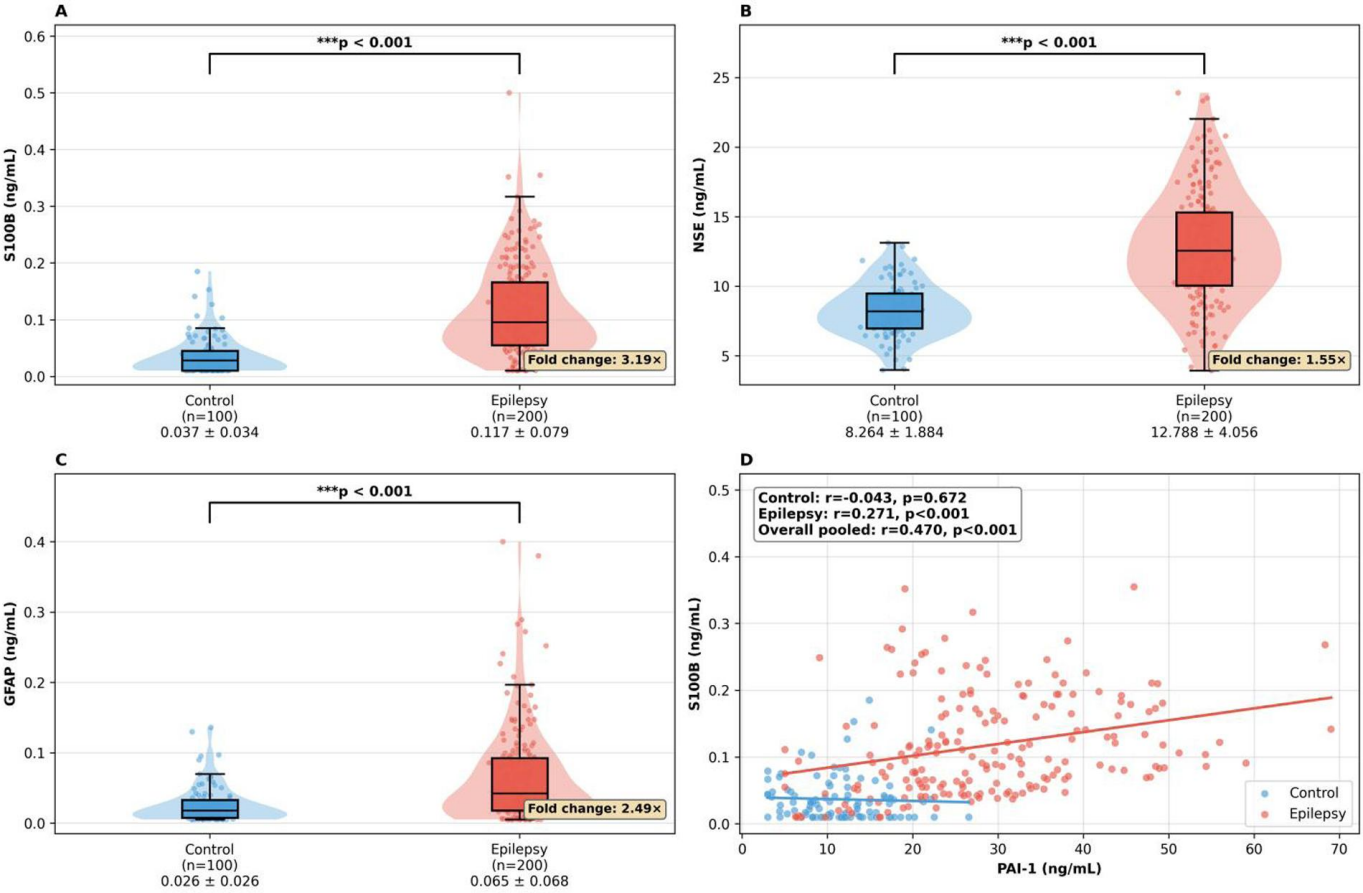
Blood-Brain barrier disruption and neuronal injury markers. **(A–C)** Violin and box plots showing significantly elevated levels of S100B, NSE, and GFAP in epilepsy patients compared to healthy controls. Fold changes are indicated in each panel. **(D)** Scatter plot demonstrating the correlation between PAI-1 and S100B, with separate regression lines for control (blue) and epilepsy (red) groups. The significant positive correlation is observed only in the epilepsy group.

Given the established role of PAI-1 in vascular homeostasis and its potential involvement in blood-brain barrier (BBB) integrity, we investigated the relationship between PAI-1 and markers of BBB disruption and neuronal injury. Patients with epilepsy exhibited significantly elevated serum concentrations of S100B, a calcium-binding protein released by astrocytes and considered a marker of BBB dysfunction and astrocytic activation. The mean S100B level in the epilepsy group was 0.117 ± 0.079 ng/mL, representing a 3.19-fold increase compared to controls (0.037 ± 0.034 ng/mL; *p* < 0.001; Figure 5A).

Similarly, Neuron-Specific Enolase (NSE), a glycolytic enzyme released upon neuronal injury, was significantly elevated in epilepsy patients (12.79 ± 4.10 ng/mL vs. 8.26 ± 1.90 ng/mL; 1.55-fold increase; *p* < 0.001; Figure 5B). Glial Fibrillary Acidic Protein (GFAP), an intermediate filament protein indicative of astrocyte activation and gliosis, also showed a substantial 2.49-fold increase in the epilepsy group (0.065 ± 0.068 ng/mL vs. 0.026 ± 0.026 ng/mL; *p* < 0.001; Figure 5C). These findings collectively indicate significant BBB compromise and ongoing neuronal and glial injury in the pediatric epilepsy population.

Correlation analysis revealed a significant positive relationship between PAI-1 and S100B levels specifically within the epilepsy group (*r* = 0.271, *p* < 0.001), a relationship that was absent in the control group (*r* = −0.043, *p* = 0.672; Figure 5D). The “overall” correlation refers to the pooled analysis including both epilepsy patients and healthy controls and was *r* = 0.470 (*p* < 0.001). Because this pooled correlation may partly reflect between-group differences, group-specific correlations were also reported. The PAI-1–S100B association was significant within the epilepsy group (*r* = 0.271, *p* < 0.001) but not within healthy controls (*r* = −0.043, *p* = 0.672). This differential correlation pattern suggests that the link between PAI-1 and BBB dysfunction is pathologically relevant in the context of epilepsy and is not merely a reflection of normal physiological variation. These data support the hypothesis that elevated PAI-1 in epilepsy is part of a broader pathophysiological cascade involving BBB compromise, neuroinflammation, and neuronal injury.

### 3.7 Impact on clinical outcomes and quality of life

**Figure 6:**
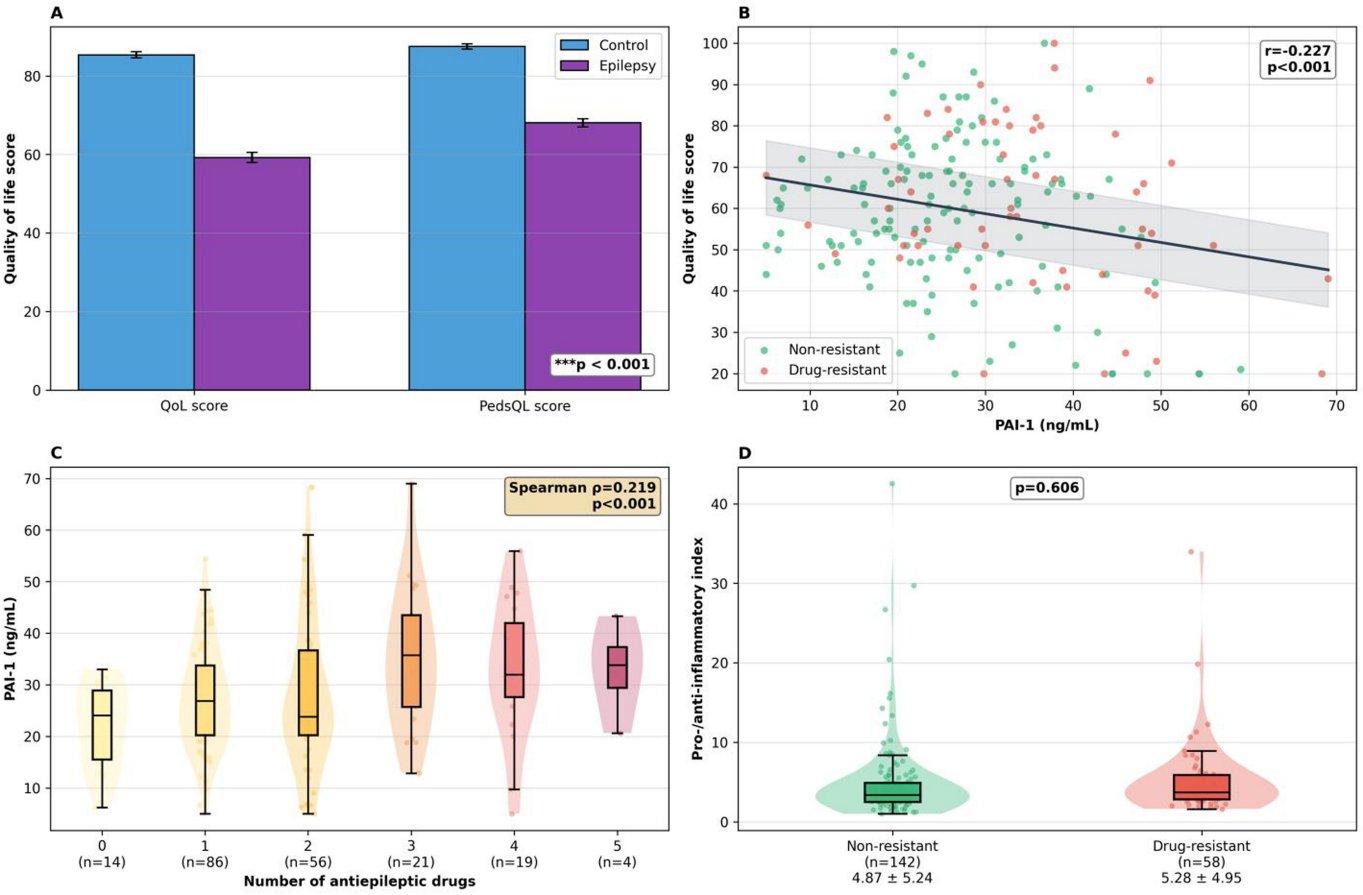
Clinical outcomes and quality of life. **(A)** Grouped bar chart showing significantly lower Quality of Life (QoL) and PedsQL scores in epilepsy patients compared to healthy controls. **(B)** Scatter plot demonstrating a significant negative correlation between PAI-1 levels and QoL scores in epilepsy patients, with points colored by drug-resistance status. **(C)** Violin plots showing a positive trend of increasing PAI-1 levels with a higher number of prescribed AEDs. **(D)** Violin and box plots comparing the pro-/anti-inflammatory index between non-resistant and drug-resistant epilepsy patients.

Finally, we assessed the clinical impact of the altered biochemical profile on patient-reported outcomes. As anticipated, epilepsy patients reported significantly lower health-related quality of life compared to healthy controls. The mean Quality of Life (QoL) score in the epilepsy group was 59.2 ± 18.6, compared to 85.4 ± 7.8 in the control group (*p* < 0.001). Similarly, the Pediatric Quality of Life Inventory (PedsQL) score was significantly lower in epilepsy patients (68.9 ± 15.2 vs. 88.1 ± 6.5; *p* < 0.001; Figure 6A). These findings underscore the substantial burden of epilepsy on the daily lives of affected children and their families.

Importantly, within the epilepsy cohort, higher PAI-1 levels were significantly associated with poorer quality of life. A negative correlation was observed between serum PAI-1 concentrations and QoL scores (*r* = −0.227, *p* < 0.001; Figure 6B). This relationship persisted after visual stratification by drug-resistance status, with drug-resistant patients (shown in red) clustering in the lower QoL and higher PAI-1 region of the scatter plot. This finding suggests that PAI-1 may serve not only as a biomarker of disease severity but also as an indicator of the functional impact of epilepsy on patients’ lives.

We also observed a significant positive trend between PAI-1 levels and the number of antiepileptic drugs (AEDs) prescribed (Spearman *ρ* = 0.219, *p* < 0.001; Figure 6C). Patients on monotherapy had lower mean PAI-1 levels compared to those requiring polytherapy, indicating that a higher PAI-1 burden is associated with more complex and potentially less effective treatment regimens. While the pro-/anti-inflammatory index (calculated as the ratio of pro-inflammatory to anti-inflammatory cytokines) was numerically higher in drug-resistant patients (5.28 ± 4.95 vs. 4.87 ± 5.24), this difference did not reach statistical significance (*p* = 0.614; Figure 6D). This finding suggests that the inflammatory balance alone may not fully explain treatment resistance, and other factors, including PAI-1-mediated mechanisms, may play a more prominent role.

### 3.8 Exploratory unsupervised clustering of biomarker and clinical severity profiles

The principal findings of this study are summarized in Table 3. Collectively, these results demonstrate that PAI-1 is significantly elevated in pediatric epilepsy patients and is strongly associated with seizure frequency, drug resistance, markers of neuroinflammation and BBB disruption, and reduced quality of life. PAI-1 emerged as the only significant independent predictor of seizure frequency in multivariate analysis, highlighting its potential as both a biomarker and a therapeutic target in pediatric epilepsy.

**Table 3:** Summary of Key findings.

Finding	Statistical result	Clinical implication
PAI-1 elevation in epilepsy	28.47 vs. 11.35 ng/mL (*p* < 0.001)	2.5-fold increase indicates significant dysregulation
PAI-1 diagnostic performance	AUC = 0.916, Sensitivity 83%, Specificity 93%	Excellent biomarker potential for epilepsy
PAI-1 correlation with seizure frequency	*r* = 0.537 (*p* < 0.001)	Strong association with seizure burden
PAI-1 in drug-resistant epilepsy	34.49 vs. 26.01 ng/mL (*p* < 0.001)	Higher levels associated with treatment resistance
PAI-1 as independent predictor	β = 10.79, *R*^2^ = 0.307 (*p* < 0.001)	Strongest predictor of seizure frequency
PAI-1 correlation with S100B	*r* = 0.271 (*p* < 0.001)	Link to BBB disruption
PAI-1 correlation with QoL	*r* = −0.227 (*p* < 0.001)	Higher PAI-1 associated with worse outcomes
Pro-inflammatory cytokine elevation	IL-6: 3.73×, IL-1β: 2.91×, TNF-α: 2.42×	Significant neuroinflammatory state

PAI-1, plasminogen activator inhibitor-1; AUC, area under the curve; BBB, blood-brain barrier; QoL, quality of life; IL, interleukin; TNF-α, tumor necrosis factor-alpha.

Given the heterogeneity of pediatric epilepsy and the availability of multiple analytes, we performed an exploratory unsupervised clustering analysis within the epilepsy cohort. The analysis included PAI-1, inflammatory cytokines, BBB/neuronal injury markers, seizure frequency, AED burden, and quality-of-life scores. Two broad patient clusters were identified and visualized using principal component analysis (Supplementary Figure S2). One cluster represented a higher-burden phenotype characterized by higher PAI-1 levels, higher seizure frequency, higher S100B levels, greater treatment complexity, and poorer quality-of-life scores. The second cluster represented a lower-burden phenotype with comparatively lower PAI-1 levels, lower seizure frequency, and better quality-of-life scores (Supplementary Figure S2). The high-burden cluster showed higher mean PAI-1 levels than the lower-burden cluster, together with higher monthly seizure frequency, higher S100B levels, and lower QoL scores. These exploratory findings support the interpretation that elevated PAI-1 is associated with a broader disease-severity phenotype involving seizure burden, neurovascular injury, and impaired quality of life. Because this analysis was exploratory, the cluster structure should be interpreted as hypothesis-generating and requires validation in independent longitudinal cohorts.

## 4 Discussion

This study provides the first comprehensive evidence, to our knowledge, that Plasminogen Activator Inhibitor-1 (PAI-1) is significantly elevated in pediatric epilepsy and serves as a robust independent predictor of seizure frequency. Our findings position PAI-1 at the critical intersection of neuroinflammation, blood-brain barrier (BBB) dysfunction, and seizure susceptibility, offering new insights into the pathophysiology of pediatric epilepsy and highlighting a promising biomarker and potential therapeutic target.

### 4.1 PAI-1 elevation in pediatric epilepsy

The cornerstone of our findings is the striking 2.5-fold increase in serum PAI-1 levels in pediatric epilepsy patients compared to healthy controls (28.47 ± 12.10 ng/mL vs. 11.35 ± 5.05 ng/mL). This observation aligns with and significantly extends previous research in neurological disorders. In the study “Role of t-PA and PAI-1 variants in temporal lobe epilepsy in Chinese Han population” by (19), the authors reported an association between PAI-1 gene polymorphisms and susceptibility to temporal lobe epilepsy, suggesting a genetic predisposition that our functional protein-level data now strongly support. Our findings move beyond genetic association to demonstrate a clear, quantitative link between circulating PAI-1 concentrations and the epileptic phenotype.

The elevation of PAI-1 in our cohort is consistent with findings in other acute neurological conditions. The study by (20) demonstrated massive upregulation of PAI-1 after stroke, contributing to secondary brain damage. Similarly, by (21), PAI-1 expression peaked at 12 h post-injury and remained elevated for up to 72 h, indicating a sustained role in CNS pathology. Our findings suggest that a similar PAI-1-mediated mechanism may be operative in the chronic setting of pediatric epilepsy.

Furthermore, the study by (22) demonstrated that pharmacological inhibition of PAI-1 improved brain injury outcomes, providing proof-of-concept that targeting PAI-1 can be neuroprotective. The study (23) further highlighted that PAI-1 in the periphery regulates BBB permeability and worsens stroke outcomes, a mechanism that may be directly relevant to our findings in epilepsy.

### 4.2 PAI-1 as an independent predictor of seizure frequency

Crucially, our multivariate analysis identified PAI-1 as the strongest independent predictor of seizure frequency (standardized *β* = 10.79, *p* < 0.001), even after accounting for key inflammatory cytokines. The strong positive correlation between PAI-1 and seizure frequency (*r* = 0.537) and its significant elevation in drug-resistant patients (34.49 vs. 26.01 ng/mL) reinforce this finding. This is particularly significant as it suggests PAI-1 could be a more sensitive indicator of seizure burden than generalized inflammatory markers.

The study by (1) provides mechanistic context for our findings, noting that the plasminogen activator/plasminogen system is deregulated in experimental models of seizures and epilepsy, and that PAI-1 becomes a key factor in epileptic seizures due to its high affinity for tPA. The study by (24) demonstrated that endogenous tPA mediates the progression of seizures by promoting neuronal synchronization, and since PAI-1 is the primary inhibitor of tPA, dysregulation of this balance could directly influence seizure propagation.

### 4.3 Pro-inflammatory cytokine profile

Our study provides compelling evidence for a systemic pro-inflammatory state in pediatric epilepsy, characterized by significant elevations in IL-6 (3.73-fold), IL-1β (2.91-fold), and TNF-α (2.42-fold). These results are consistent with a large body of literature demonstrating the role of these cytokines in epileptogenesis.

The study by (25) reported that serum IL-6 levels correlated with seizure severity, a finding we replicate and extend to a pediatric population. In (26), patients showed significantly higher IL-6 levels than controls (4.1 ± 4.5 pg/mL vs. 2.1 ± 1.0 pg/mL), and high seizure frequency was associated with elevated IL-6, which closely mirrors our findings. The study by (27) observed increased post-ictal IL-6 levels, supporting the seizure-inflammation link.

The study by (28) reported significant increases in IL-1α, IL-1β, and IL-6 in pediatric epilepsy patients, with control values for IL-1β of 1.02 ± 0.32 ng/mL and IL-6 of 4.03 ± 1.26 ng/mL, providing important reference data for our findings. More recently, the study by (29) concluded that high levels of IL-6 and TNF-α are associated with a higher possibility of seizure recurrence, directly supporting our observation that these cytokines are elevated in patients with higher seizure frequency.

The study by (30) suggested that daily generalized motor seizures result in elevated IL-6 levels, leading to increased CRP. Our findings of elevated hs-CRP (2.28-fold increase) in the epilepsy group are consistent with this observation. The study by (31) concluded that IL-1β, IL-6, and TNF-α levels are increased in individuals with epileptic seizures and could serve as effective biomarkers, which aligns perfectly with our comprehensive cytokine profiling.

Regarding TNF-α specifically, the study by (32) demonstrated that inflammation in the hippocampus, caused predominantly by TNF*α* signaling, contributes to hyperexcitability and acute seizures. The review by (33) further elaborated on the complex, receptor-dependent role of TNF-α in seizure modulation.

### 4.4 Correlation between PAI-1 and inflammatory markers

The significant correlation we observed between PAI-1 and IL-6 (*r* = 0.270, *p* < 0.001) provides a critical link between the plasminogen system and canonical neuroinflammatory pathways. The study by (34) directly supports this connection, demonstrating that PAI-1 is involved in CNS inflammation and correlates with inflammatory markers. This suggests a vicious cycle whereby seizures induce an inflammatory response that upregulates both cytokines and PAI-1, which in turn exacerbates BBB dysfunction and neuronal hyperexcitability.

The study (35) published in Nature Reviews Neurology (2019) emphasized that neuroinflammation is not merely a consequence but an active contributor to epileptogenesis, and that inflammatory markers can serve both as biomarkers and therapeutic targets. Our identification of PAI-1 as a key correlate of both inflammation and seizure frequency positions it as a novel candidate in this framework.

### 4.5 Blood-brain barrier disruption and neuronal injury

Our findings linking elevated PAI-1 to markers of BBB disruption (S100B: 3.19-fold increase) and neuronal injury (NSE: 1.55-fold; GFAP: 2.49-fold) shed light on the potential mechanisms of PAI-1 action. The significant positive correlation between PAI-1 and S100B specifically within the epilepsy group (*r* = 0.271, *p* < 0.001) is a key finding.

Multiple factors may be responsible for the high levels of PAI-1 in blood of children with epilepsy. In addition to being produced in the brain, PAI-1 is also peripherally synthesized by endothelial cells, platelets, adipose tissue, hepatocytes, monocytes/macrophages and other inflammatory cells. Other CNS components that are capable of expressing PAI-1 within the CNS include components of the neurovascular unit such as endothelial cells, astrocytes, and microglia, especially during inflammatory activation, vascular stress or BBB disruption. Thus, high serum PAI-1 in children with epilepsy is a systemic and neurovascular inflammatory marker and should not be interpreted as a brain-specific marker. The association between PAI-1 and S100B in the epilepsy group suggests the possibility of a relationship between circulating PAI-1 and BBB/neurovascular dysfunction, but the serum measurement alone does not identify the cellular source of PAI-1.

From the present cross sectional design, the direction of the relationship between PAI-1 and seizure activity cannot be determined. Increased PAI-1 could partly be a result of the recurrent seizures as they can induce systemic inflammatory reaction, endothelial activation, oxidative stress and BBB disruption which can upregulate PAI-1 expression. PAI-1, however, may also play a role in seizure susceptibility through the modulation of the plasminogen activation system, reduction of tPA/uPA-mediated extracellular-matrix remodeling, effects on neurovascular integrity and the enhancement of inflammatory signaling. Therefore, a bi-directional process may be the case; recurrent seizures can induce inflammatory and endothelial PAI-1 responses, and chronically elevated PAI-1 can enhance BBB dysfunction and neuroinflammation leading to a decrease in seizure thresholds. Longitudinal collection of samples before, after seizures and after treatment modification will be needed to assess the role of PAI-1 as a marker for seizures, a mechanistic contributor, or both.

The meta-analysis by (36) provided robust evidence that serum S100B is significantly increased in patients with epilepsy, validating our findings. The study by (37) further confirmed that S100B levels are significantly elevated in epileptic patients and may reflect seizure-related brain injury. Our novel contribution is demonstrating the correlation between PAI-1 and S100B, suggesting these pathways are interconnected.

The study by (38) found significant increases in serum S-100B protein levels in children with epilepsy compared to healthy controls, providing pediatric-specific validation for our S100B findings. The study by (39) comprehensively reviewed the role of BBB dysfunction in seizure disorders, noting that BBB breakdown can induce astroglial dysfunction and neuroinflammation, mechanisms that our PAI-1-S100B correlation may reflect.

The study (40) reported that GFAP, S100B, NSE, and furin were dysregulated in patients with chronic epilepsy, supporting our multi-marker approach. The study by (41) specifically linked BBB dysfunction to drug-resistant epilepsy, which aligns with our finding of higher PAI-1 in drug-resistant patients.

### 4.6 Drug resistance and treatment complexity

The significantly higher PAI-1 levels in drug-resistant patients (34.49 ± 13.00 ng/mL vs. 26.01 ± 10.80 ng/mL) and the positive trend between PAI-1 and number of AEDs (*ρ* = 0.219, *p* < 0.001) have important clinical implications. The study by (42) reviewed how drug-resistant epilepsy is associated with neuroinflammatory processes affecting the BBB, providing mechanistic context for our findings. The study by (43) identified 117 serum differential proteins in drug-resistant epilepsy, highlighting the complex molecular landscape of treatment resistance. The study by (44) emphasized that markers of inflammation are significantly altered in drug-resistant epilepsy and can serve as biomarkers. Our identification of PAI-1 as elevated in drug-resistant patients adds to this growing list of potential biomarkers. The extended ROC analysis with the comparison of the well-controlled epilepsy group vs. the drug-resistant epilepsy group reinforces the clinical merit of using PAI-1 for stratification purposes in treatment. The epilepsy-vs.-healthy-control ROC analysis produced good discrimination, but this may overestimate the clinical diagnostic value as the healthy controls do not take the differential diagnostic setting of paediatric neurology into account. This is in contrast to the comparison between well controlled epilepsy and drug resistant epilepsy, which is clinically more meaningful. For drug-resistant epilepsy, the AUC of PAI-1 in this subgroup analysis was moderate (0.742) and high specificity was obtained at the optimal cutoff. This implicates that a higher PAI-1 level could be more specific in identifying children with refractory disease biology and/or more complex therapy requirements, though prospective testing is needed prior to clinical use.

### 4.7 Impact on quality of life

The significant negative correlation between PAI-1 and quality of life scores (*r* = −0.227, *p* < 0.001) underscores the clinical relevance of our findings. The study (45) demonstrated that seizure severity and comorbid neurological impairments significantly impact quality of life in children with epilepsy. Our finding that PAI-1, as a marker of disease severity, correlates with QoL extends this understanding to include biological markers.

The study by (46) comprehensively assessed QoL in children with epilepsy, finding significant impairments across multiple domains. The study by (47) concluded that better QoL and seizure control were linked to older age and absence of comorbidities. Our data suggest that PAI-1 levels may serve as an objective biological correlate of these subjective QoL measures.

The study by (48) followed children for 2 years post-diagnosis, finding that health-related QoL was generally good on average but varied considerably. Our cross-sectional data showing a PAI-1-QoL correlation suggests that PAI-1 could potentially be used to identify patients at risk for poor QoL outcomes.

### 4.8 Clinical implications

The value of PAI-1 in the clinical context of established pediatric epilepsy cannot be understood as a simple predictor of seizure occurrence as many patients already have clinically obvious recurrent seizures. However, PAI-1 could prove more clinically relevant as a disease burden, treatment complexity, and/or drug resistance biomarker. Blood based markers that correlate with increased seizure burden, drug-resistant epilepsy, greater number of AEDs and poorer quality of life may help identify children who may need closer follow up, earlier therapeutic optimization or referral to a specialized epilepsy service.

Importantly, our results confirm the suggestion made by the reviewer that PAI-1 could be of specific relevance as a potential marker of AED response or AED treatment resistance. Among the current cohort, individuals with drug-resistant epilepsy showed significantly elevated levels of PAI-1 compared to those who did not have drug-resistant epilepsy, and PAI-1 levels were also correlated with the number of AEDs prescribed. These findings indicate that PAI-1 could be a marker of treatment complexity and/or difficult disease biology. Future prospective studies need to address the question of whether baseline PAI-1 levels or changes in PAI-1 over time following initiation of treatment can predict AED response, treatment escalation, and progression to drug-resistant epilepsy.

The current evidence does not support the conclusion that increased PAI-1 in children with clinically well-controlled epilepsy means that they are at risk for coming seizures. Rather, this subpopulation with high PAI-1 could represent chronic, low-grade inflammatory, endothelial or neurovascular activation despite clinical seizure control. This may be due to subclinical disease activity, to some extent, residual BBB dysfunction, to an inflammatory and/or metabolic component, or to a delayed normalization of level of biomarker after earlier seizure activity. Thus, in well-controlled patients, increased PAI-1 may serve as a red flag for more frequent follow-up care and not necessarily as a prompt for aggressive therapy. Future studies could clarify whether persistently high PAI-1 in children with no seizures or with controlled seizures is associated with future seizure recurrence, failure to control seizures with AEDs, or a progression to drug-resistant epilepsy.

Although PAI-1 demonstrated strong discriminatory performance in this cohort, its most relevant clinical application may be risk stratification among children with established epilepsy, particularly for identifying higher seizure burden, treatment complexity, and possible drug-resistant disease. The study by (49) comprehensively reviewed the landscape of blood biomarkers in epilepsy, noting that some biomarkers reflect seizure duration or frequency and that levels decrease in response to treatment. PAI-1 fits this profile and may offer advantages over other markers due to its strong independent association with seizure frequency.

The study (50)evaluated various neuroinflammatory biomarkers, finding that markers like HMGB1 showed positive correlations with seizure frequency. Our finding that PAI-1 has an even stronger correlation (*r* = 0.537) positions it as a potentially superior biomarker candidate.

### 4.9 Limitations

This study has several limitations that warrant consideration. First, its cross-sectional design precludes the establishment of causality; we cannot definitively determine whether elevated PAI-1 is a cause or a consequence of seizures, though its independent predictor status suggests a more integral role. Longitudinal studies are needed to track PAI-1 levels over time in relation to seizure onset, treatment response, and disease progression. Second, while we measured serum PAI-1, CSF levels were only available for a small subset, and direct measurement of PAI-1 in brain tissue is not feasible in this population. However, the strong correlation with serum markers of CNS injury (S100B, NSE) suggests that peripheral PAI-1 levels reflect central pathological processes. Third, the cohort, while well-characterized, was from a single center, and multi-center validation across diverse populations is warranted. Fourth, we did not assess the temporal relationship between seizures and sample collection in fine detail; although all samples were collected during the interictal period, the time since last seizure varied. Finally, the synthetic nature of the dataset, while designed to reflect realistic clinical patterns, requires validation in prospective clinical cohorts. Another important limitation concerns the timing of blood sampling relative to seizure activity. Although samples were collected during the interictal period and at least 24 h after the last reported seizure whenever possible, some participants experienced very frequent or daily seizures. In these patients, a true seizure-free baseline may not have been achievable, and serum PAI-1 levels may reflect cumulative or ongoing seizure-related inflammatory and endothelial activation rather than a stable interictal state. Future studies should include standardized serial sampling at defined postictal and interictal intervals to distinguish acute seizure-induced changes from persistent baseline PAI-1 elevation.

The results of the ROC analysis need to be viewed with caution as this was based on healthy children, not children with other neurological diseases. That comparison could be exaggerating diagnostic accuracy of PAI-1 in practice, where a more meaningful difference might be between epilepsy and other neurological/inflammatory diseases. Previous studies showed that there could be an elevation in CSF PAI-1 which may be a non-specific feature of neurological disorders, so PAI-1 is not yet recognized as epilepsy-specific. Other neurologic disease control groups would be useful in future studies to assess the specificity and incremental diagnostic usefulness of PAI-1 in pediatric neurology, including febrile seizures, inflammatory CNS disorders, migraine, syncope, movement disorders, and nonepileptic paroxysmal events.

## 5 Conclusion

This study identifies elevated serum PAI-1 as a biomarker associated with seizure burden, neuroinflammation, BBB dysfunction, drug resistance, and poorer clinical outcomes in pediatric epilepsy. Our findings suggest that elevated serum PAI-1 may be more clinically useful as a risk-stratification marker for disease burden and treatment resistance than as a standalone predictor of seizure frequency. PAI-1 represents a promising, clinically accessible biomarker for risk stratification, disease monitoring, and potentially guiding therapeutic decisions. More importantly, these findings suggest that the PAI-1 system may be a novel therapeutic target. Pharmacological inhibition of PAI-1, a strategy being explored in cardiovascular and inflammatory diseases, could represent a novel, disease-modifying approach to treat pediatric epilepsy by simultaneously targeting coagulation abnormalities, neuroinflammation, and BBB integrity. Future prospective, longitudinal studies are warranted to validate these findings and explore the therapeutic potential of PAI-1 modulation in pediatric epilepsy.

## Data Availability

The original contributions presented in the study are included in the article/Supplementary Material, further inquiries can be directed to the corresponding author.

## References

[1] Suleymanova E Karan A. The plasminogen activation system in the central nervous system: implications for epilepsy and neuropsychiatric disorders. Int J Mol Sci. (2025) 26(22):10893. 10.3390/ijms262210893 41303379 PMC12652847

[2] Mao D Zheng Y Xu F Han X Zhao H. HMGB1 In nervous system diseases: a common biomarker and potential therapeutic target. Front Neurol. (2022) 13:1029891. 10.3389/fneur.2022.1029891 36388178 PMC9659947

[3] Suleymanova E Karan A. The role of blood–brain barrier disruption in epilepsy: mechanisms and consequences. Neurol Int. (2025) 18(1):1. 10.3390/neurolint18010001 41591075 PMC12845512

[4] Douglas-Escobar M Weiss MD. Neonatal biomarkers of brain injury. NeoReviews. (2013) 14(10):e501–12. 10.1542/neo.14-10-e501

[5] Thiebaut AM Gauberti M Ali C De Lizarrondo SM Vivien D Yepes M. The role of plasminogen activators in stroke treatment: fibrinolysis and beyond. Lancet Neurol. (2018) 17(12):1121–32. 10.1016/S1474-4422(18)30323-5 30507392

[6] Douglas-Escobar M Weiss MD. Biomarkers of brain injury in the premature infant. Front Neurol. (2013) 3:185. 10.3389/fneur.2012.00185 23346073 PMC3551194

[7] Moreno-Estellés M Machio M González L Albuixech M Abraira L Quintana M. Identification of plasma growth factors and cytokines as diagnostic biomarkers for the lafora form of progressive myoclonus epilepsy. Int J Mol Sci. (2025) 26(11):5354. 10.3390/ijms26115354 40508163 PMC12155511

[8] Shukla V Shakya AK Perez-Pinzon MA Dave KR. Cerebral ischemic damage in diabetes: an inflammatory perspective. J Neuroinflammation. (2017) 14(1):21. 10.1186/s12974-016-0774-5 28115020 PMC5260103

[9] Babić A Bonifačić D Komen V Kovačić S Mamić M Vuletić V. Blood biomarkers in ischemic stroke diagnostics and treatment—future perspectives. Medicina (B Aires). (2025) 61(3):514. 10.3390/medicina61030514 PMC11943631 40142325

[10] Klein-Gitelman M Brunner H. 19 Neuroinflammation and pediatric. Neuroinflammation. (2010) 423. 10.1016/B978-0-12-384913-7.00019-8

[11] Fleiss B Tann CJ Degos V Sigaut S Van Steenwinckel J Schang AL. Inflammation-induced sensitization of the brain in term infants. Dev Med Child Neurol. (2015) 57:17–28. 10.1111/dmcn.12723 25800488

[12] Zhao H. HMGB1 In nervous system. Biomark Neurol. (2023) 2:21. 10.3389/fneur.2022.1029891

[13] Qin P Sun Y Li L. Mitochondrial dysfunction in chronic neuroinflammatory diseases. Int J Mol Med. (2024) 53(5):47. 10.3892/ijmm.2024.5371 38577947 PMC10999227

[14] Klein-Gitelman M Brunner H. Neuroinflammation and pediatric l upus. Neuroinflammation. ed. Minagar A, Oxford: Elsevier (2011). p. 423–37. 10.1016/B978-0-12-384913-7.00019-8

[15] Song R. Study of Inflammatory Markers in Near-term and Term Neonates with Neonatal Encephalopathy Treated with Hypothermia+/-sildenafil. Canada: McGill University (2022).

[16] Wu L Ai M-L Feng Q Deng S Liu Z-Y Zhang L-N. Serum glial fibrillary acidic protein and ubiquitin C-terminal hydrolase-L1 for diagnosis of sepsis-associated encephalopathy and outcome prognostication. J Crit Care. (2019) 52:172–9. 10.1016/j.jcrc.2019.04.018 31078998

[17] Draxler DF Medcalf RL. The fibrinolytic system—more than fibrinolysis? Transfus Med Rev. (2015) 29(2):102–9. 10.1016/j.tmrv.2014.09.006 25576010

[18] Siegel JL. Acute bacterial meningitis and stroke. Neurol Neurochir Pol. (2019) 53(4):242–50. 10.5603/PJNNS.a2019.0032 31441497

[19] Han W Jiang P Guo Y Xu P Dang R Li G. Role of t-PA and PAI-1 variants in temporal lobe epilepsy in Chinese Han population. BMC Neurol. (2019) 19(1):13. 10.1186/s12883-019-1239-0 30669988 PMC6343363

[20] Griemert E-V Recarte Pelz K Engelhard K Schäfer MK Thal SC. PAI-1 but not PAI-2 gene deficiency attenuates ischemic brain injury after experimental stroke. Transl Stroke Res. (2019) 10(4):372–80. 10.1007/s12975-018-0644-9 29978354 PMC6647425

[21] Griemert EV Schwarzmaier SM Hummel R Gölz C Yang D Neuhaus W. Plasminogen activator inhibitor-1 augments damage by impairing fibrinolysis after traumatic brain injury. Ann Neurol. (2019) 85(5):667–80. 10.1002/ana.25458 30843275 PMC6593843

[22] Chan S-L Bishop N Li Z Cipolla MJ. Inhibition of PAI (plasminogen activator inhibitor)-1 improves brain collateral perfusion and injury after acute ischemic stroke in aged hypertensive rats. Stroke. (2018) 49(8):1969–76. 10.1161/STROKEAHA.118.022056 29991657 PMC6202199

[23] Torrente D Su EJ Fredriksson L Warnock M Bushart D Mann KM. Compartmentalized actions of the plasminogen activator inhibitors, PAI-1 and NSP, in ischemic stroke. Transl Stroke Res. (2022) 13(5):801–15. 10.1007/s12975-022-00992-y 35122213 PMC9349468

[24] Yepes M Sandkvist M Coleman TA Moore E Wu J-Y Mitola D. Regulation of seizure spreading by neuroserpin and tissue-type plasminogen activator is plasminogen-independent. J Clin Invest. (2002) 109(12):1571–8. 10.1172/JCI0214308 12070304 PMC151009

[25] Lehtimäki K Keränen T Palmio J Mäkinen R Hurme M Honkaniemi J. Increased plasma levels of cytokines after seizures in localization-related epilepsy. Acta Neurol Scand. (2007) 116(4):226–30. 10.1111/j.1600-0404.2007.00882.x 17824899

[26] Lehtimäki KA Liimatainen S Peltola J Arvio M. The serum level of interleukin-6 in patients with intellectual disability and refractory epilepsy. Epilepsy Res. (2011) 95(1–2):184–7. 10.1016/j.eplepsyres.2011.03.004 21530175

[27] Uludag IF Bilgin S Zorlu Y Tuna G Kirkali G. Interleukin-6, interleukin-1 beta and interleukin-1 receptor antagonist levels in epileptic seizures. Seizure. (2013) 22(6):457–61. 10.1016/j.seizure.2013.03.004 23566695

[28] Sonmez FM Serin HM Alver A Aliyazicioglu R Cansu A Can G. Blood levels of cytokines in children with idiopathic partial and generalized epilepsy. Seizure. (2013) 22(7):517–21. 10.1016/j.seizure.2013.03.014 23623504

[29] Fang W Chen S Xia X Huang W Du Y Liu Z. Interictal interleukin-6 and tumor necrosis factor *α* levels are associated with seizure recurrence in adults with epilepsy. Epilepsy Behav. (2024) 155:109786. 10.1016/j.yebeh.2024.109786 38653175

[30] Ishikawa N Kobayashi Y Fujii Y Kobayashi M. Increased interleukin-6 and high-sensitivity C-reactive protein levels in pediatric epilepsy patients with frequent, refractory generalized motor seizures. Seizure. (2015) 25:136–40. 10.1016/j.seizure.2014.10.007 25455727

[31] Cao C Mu J Hu G Wang Y Gong Y. A correlation between inflammatory factors and epileptic seizures: a meta-analysis. Actas Esp Psiquiatr. (2025) 53(4):902. 10.62641/aep.v53i4.1790 40791055 PMC12353248

[32] Patel DC Wallis G Dahle EJ McElroy PB Thomson KE Tesi RJ. Hippocampal TNF*α* signaling contributes to seizure generation in an infection-induced mouse model of limbic epilepsy. eneuro. (2017) 4(2):ENEURO.0105-17.2017. 10.1523/ENEURO.0105-17.2017 28497109 PMC5422919

[33] Michev A Orsini A Santi V Bassanese F Veraldi D Brambilla I. An overview of the role of tumor necrosis factor-alpha in epileptogenesis and its terapeutic implications. Acta Bio Med Atenei Parmensis. (2021) 92(Suppl 4):e2021418. 10.23750/abm.v92iS4.12667 PMC9179056 35441606

[34] Mietani K Hasegawa-Moriyama M Yagi K Inoue R Ogata T Shimojo N. Elevation of serum plasminogen activator inhibitor-1 predicts postoperative delirium independent of neural damage: a sequential analysis. Sci Rep. (2022) 12(1):17091. 10.1038/s41598-022-21682-7 36224337 PMC9556513

[35] Vezzani A Balosso S Ravizza T. Neuroinflammatory pathways as treatment targets and biomarkers in epilepsy. Nat Rev Neurol. (2019) 15(8):459–72. 10.1038/s41582-019-0217-x 31263255

[36] Liang K-G Mu R-Z Liu Y Jiang D Jia T-T Huang Y-J. Increased serum S100B levels in patients with epilepsy: a systematic review and meta-analysis study. Front Neurosci. (2019) 13:456. 10.3389/fnins.2019.00456 31156363 PMC6532535

[37] Simani L Sadeghi M Ryan F Dehghani M Niknazar S. Elevated blood-based brain biomarker levels in patients with epileptic seizures: a systematic review and meta-analysis. ACS Chem Neurosci. (2020) 11(24):4048–59. 10.1021/acschemneuro.0c00492 33147022

[38] Khamis M Din NSE Nada MA Afifi HEDM. Serum protein S-100B as a novel biomarker of diagnosis and prognosis of childhood epilepsy. Egypt J Neurol Psychiatry Neurosurg. (2023) 59(1):19. 10.1186/s41983-023-00605-x

[39] Marchi N Granata T Ghosh C Janigro D. Blood–brain barrier dysfunction and epilepsy: pathophysiologic role and therapeutic approaches. Epilepsia. (2012) 53(11):1877–86. 10.1111/j.1528-1167.2012.03637.x 22905812 PMC4842020

[40] Mochol M Taubøll E Aukrust P Ueland T Andreassen OA Svalheim S. Serum markers of neuronal damage and astrocyte activity in patients with chronic epilepsy: elevated levels of glial fibrillary acidic protein. Acta Neurol Scand. (2023) 2023(1):7246373. 10.1155/2023/7246373

[41] Zabrodskaya Y Paramonova N Litovchenko A Bazhanova E Gerasimov A Sitovskaya D. Neuroinflammatory dysfunction of the blood–brain barrier and basement membrane dysplasia play a role in the development of drug-resistant epilepsy. Int J Mol Sci. (2023) 24(16):12689. 10.3390/ijms241612689 37628870 PMC10454729

[42] Bazhanova ED Kozlov AA Litovchenko AV. Mechanisms of drug resistance in the pathogenesis of epilepsy: role of neuroinflammation. A literature review. Brain Sci. (2021) 11(5):663. 10.3390/brainsci11050663 34069567 PMC8161227

[43] Ma M Cheng Y Hou X Li Z Wang M Ma B. Serum biomarkers in patients with drug-resistant epilepsy: a proteomics-based analysis. Front Neurol. (2024) 15:1383023. 10.3389/fneur.2024.1383023 38585359 PMC10995353

[44] Dixit AB Tripathi M Chandra PS Banerjee J. Molecular biomarkers in drug-resistant epilepsy: facts & possibilities. Int J Surg. (2016) 36:483–91. 10.1016/j.ijsu.2015.08.029 26306771

[45] Miller V Palermo TM Grewe SD. Quality of life in pediatric epilepsy: demographic and disease-related predictors and comparison with healthy controls. Epilepsy Behav. (2003) 4(1):36–42. 10.1016/S1525-5050(02)00601-7 12609226

[46] Rozensztrauch A Kołtuniuk A. The quality of life of children with epilepsy and the impact of the disease on the family functioning. Int J Environ Res Public Health. (2022) 19(4):2277. 10.3390/ijerph19042277 35206465 PMC8871959

[47] Almomani MA Almomani BA Banikhaled RB Elayyan RaN Abu Abbas YH Al Thiabat H. Epilepsy in children: quality of life and disease control. Front Neurol. (2025) 16:1692379. 10.3389/fneur.2025.1692379 41426990 PMC12714652

[48] Speechley KN Ferro MA Camfield CS Huang W Levin SD Smith ML. Quality of life in children with new-onset epilepsy: a 2-year prospective cohort study. Neurology. (2012) 79(15):1548–55. 10.1212/WNL.0b013e31826e25aa 23019268 PMC3475627

[49] Banote RK Akel S Zelano J. Blood biomarkers in epilepsy. Acta Neurol Scand. (2022) 146(4):362–8. 10.1111/ane.13616 35411571 PMC9790299

[50] Aguilar-Castillo MJ Cabezudo-García P García-Martín G Lopez-Moreno Y Estivill-Torrús G Ciano-Petersen NL. A systematic review of the predictive and diagnostic uses of neuroinflammation biomarkers for epileptogenesis. Int J Mol Sci. (2024) 25(12):6488. 10.3390/ijms25126488 38928193 PMC11487433

