# Supplementary figures and images for: Elevated serum plasminogen activator inhibitor-1 is associated with seizure burden, drug resistance, and neuroinflammatory markers in pediatric epilepsy
Source: Front Pediatr. 2026 Jul 14;14:1804857. doi: 10.3389/fped.2026.1804857 (PMC13407985; doi:10.3389/fped.2026.1804857)

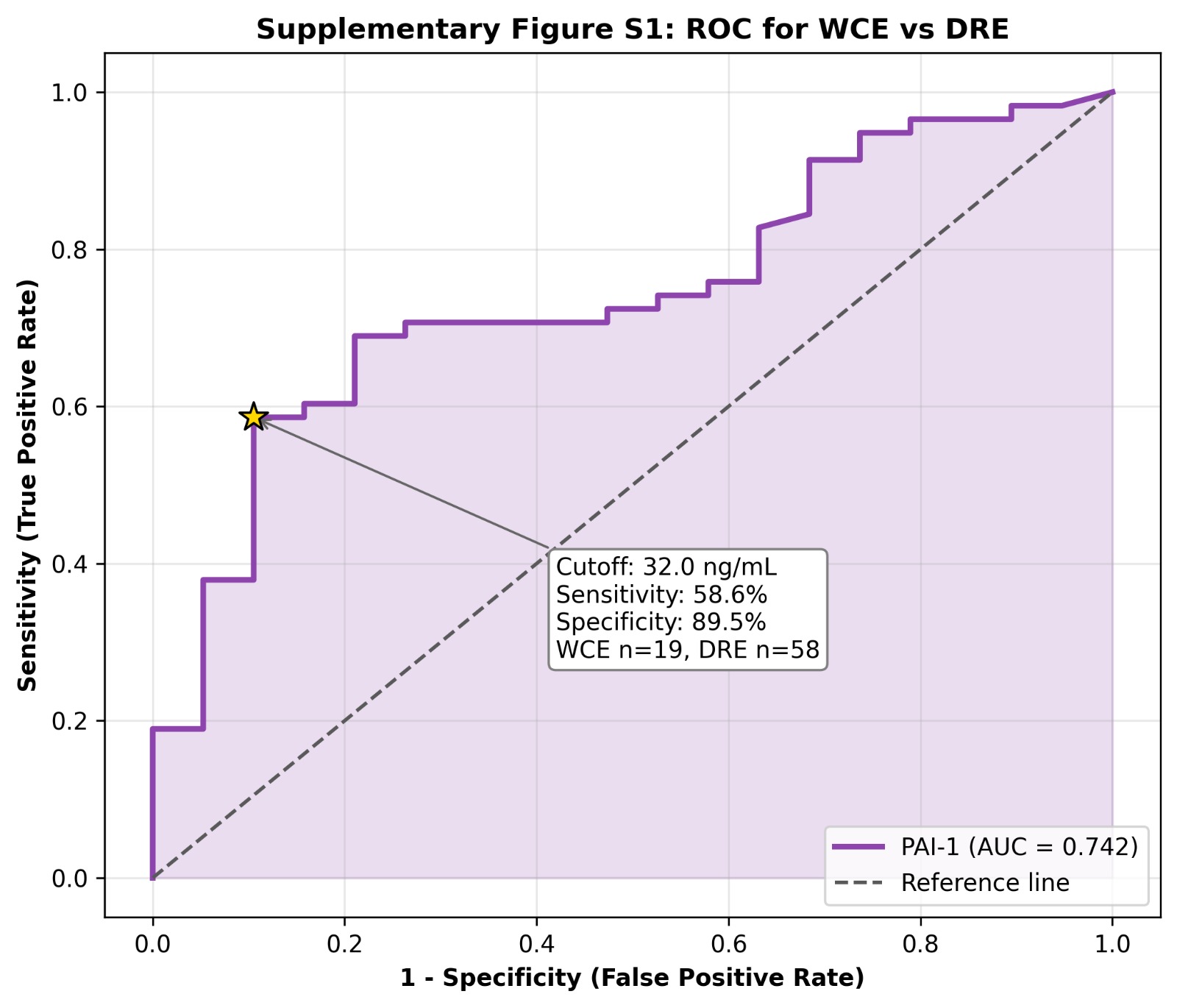

Supplement: Supplementary file 1 [file Image1.jpeg]

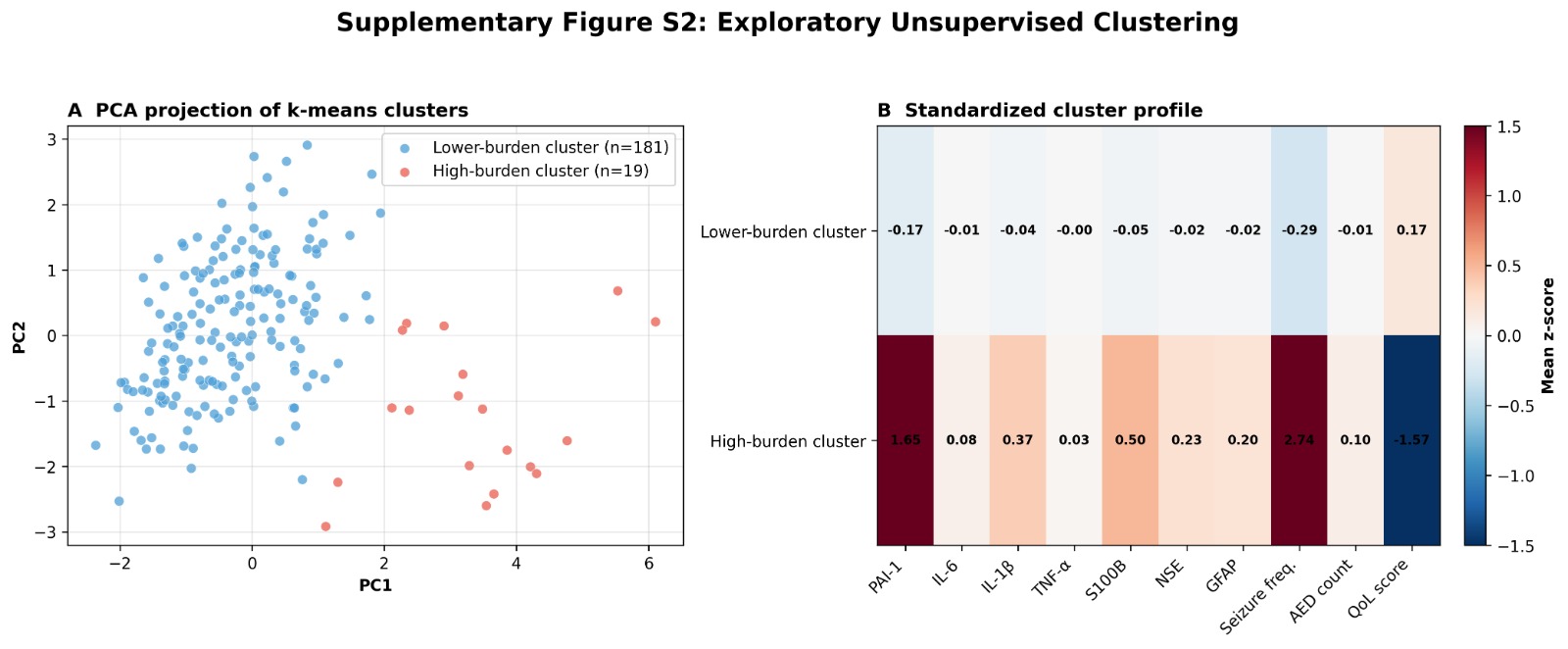

Supplement: Supplementary file 2 [file Image2.jpeg]
